# Krill Oil for Pain in Elders: protocol for a pilot, double-blind, randomised controlled trial to evaluate the efficacy of 4 g/d krill oil supplementation for chronic musculoskeletal pain and mobility limitations in older adults

**DOI:** 10.1017/S0007114526107557

**Published:** 2026-05-18

**Authors:** Javier A. Tamargo, Katarina Simic, Samira Capote, Rene Przkora, Kimberly T. Sibille, Yenisel Cruz-Almeida, Steve D. Anton

**Affiliations:** 1 Pain Research and Intervention Center of Excellence, University of Florida, USA; 2 Claude D. Pepper Older Americans Independence Center, University of Florida, USA; 3 Department of Community Dentistry and Behavioral Science, University of Florida, USA; 4 Department of Anesthesiology, University of Florida, USA; 5 Department of Physical Medicine & Rehabilitation, University of Florida, USA; 6 Department of Physiology and Aging, University of Florida, USA

**Keywords:** Chronic pain, Omega-3 fatty acids, Nutritional supplementation, Aging, Physical function

## Abstract

Mobility limitations due to chronic musculoskeletal pain are a major contributor to disability in older adults, yet current pharmacological treatments often have limited efficacy and increase the risk of polypharmacy. Omega (ω)-3 PUFA, particularly EPA and DHA, have demonstrated anti-inflammatory and analgesic properties, but are under-consumed among older USA adults. Krill oil, a marine-derived source of EPA and DHA with enhanced bioavailability compared with typical fish oils and additional bioactive compounds such as astaxanthin and choline, may offer a promising nutritional intervention. This pilot study will assess the feasibility and acceptability of a 3-month randomised, double-blind, placebo-controlled trial of krill oil supplementation (4 g/d: 1288 mg EPA + DHA, 0·45 mg astaxanthin, 320 mg choline) *v*. placebo (mixed vegetable oils) in forty community-dwelling adults aged ≥ 60 years with chronic musculoskeletal pain. Primary outcomes include feasibility (recruitment, retention and adherence) and acceptability (participant satisfaction). Secondary outcomes include changes in the omega-3 index, ω-6/ω-3 ratio and inflammation (high-sensitivity C-reactive protein), as well as exploratory changes in pain intensity and functional interference, and physical function (Short Physical Performance Battery, 6-Minute Walk Test). Findings will inform the design of future fully powered trials that may ultimately contribute to the evidence for omega-3 supplementation as a non-pharmacological strategy to support healthy aging and functional independence in older adults.

Mobility limitations affect approximately a third of older adults in the USA^(1)^, significantly reducing their independence and quality of life^(2)^. Considered a key hallmark of functional aging^(3)^, mobility limitations are indicative of physical decline, predicting disability and mortality^(4,5)^. Chronic musculoskeletal pain^(6)^, which is similarly prevalent among older adults^(7)^, contributes significantly to mobility limitations^(8,9)^ and functional decline^(10–12)^ and is the greatest cause of disability worldwide^(13)^. With the proportion of older adults projected to comprise nearly a quarter of the USA population by 2060^(14)^, the burden of chronic pain and its impact on physical function represent urgent challenges to public health and health care systems.

Managing chronic pain and preventing functional decline in older adults remain significant clinical challenges^(15–18)^. Traditional pharmacological treatments for chronic pain typically include non-opioid analgesics (e.g. nonsteroidal anti-inflammatory drugs (NSAID)) for mild to moderate pain and opioids for moderate to severe pain^(18)^. While these treatments may offer some benefit, they contribute to increased risks in older adults due to age-related changes in drug metabolism and polypharmacy^(15,18)^. Non-pharmacological approaches for pain and physical function in older adults are urgently needed^(7,15)^.

Considered generally safe and cost-effective, supplementation with omega (ω)-3 PUFA has been proposed for various age-related conditions, including chronic pain, cognitive health and functional decline^(19–21)^. Despite being essential nutrients, ω-3s are inadequately consumed by most older USA adults^(22–24)^. Omega-3 PUFA, particularly EPA and DHA, are recognised for their anti-inflammatory properties and potential health benefits in aging^(20)^. Omega-3s could also reduce neuropathic and nociceptive pain by modulating peripheral and central sensitisation^(25,26)^. Additionally, ω-3s may support physical function. Recent meta-analyses of ω-3 supplementation trials have shown improvements in lower-body strength and function in older adults^(27,28)^.

Both ω-6 and ω-3 PUFA are essential nutrients with beneficial properties as compared with saturated fats. Due to a competing pathway of long-chain PUFA synthesis enzymes, an imbalance of ω-6 to ω-3 PUFA results in the overproduction of pro-inflammatory signalling molecules. As such, an ω-6:ω-3 ratio of 4:1 to 1:1 has been considered optimal for human health; yet, the typical Western diet has resulted in ratios of 15:1 or greater^(29)^. An elevated ω-6:ω-3 ratio is associated with chronic pain prevalence and increased pain severity^(30,31)^. Moreover, low serum ω-3 is associated with reduced muscle strength, slower gait speed and mobility disability among older adults^(32–34)^. Given the abundance of ω-6s in the Western diet and poor consumption of ω-3s, supplementation with ω-3 PUFA is now thought to be essential^(29)^.

Krill oil, derived from *Euphausia superba*, has been proposed as an advantageous alternative to traditional fish oils due to greater bioavailability^(35)^. Indeed, in healthy volunteers, comparable rises in plasma ω-3 levels were achieved with krill oil and fish oil supplementation, despite a nearly 30 % lower EPA + DHA content in krill oil^(36)^. When similar EPA + DHA contents are provided, krill oil demonstrates a higher 72-hour bioavailability compared with fish oil^(37)^. However, because the EPA and DHA content per gram is typically lower in krill oil than that of standard fish oils, higher volumes of krill oil (e.g. number of capsules) may be required to achieve target EPA + DHA intake levels. As dosing recommendations remain based on absolute intake, the potential need for higher volumes may offset the bioavailability advantage and may influence dosing considerations and acceptability, particularly among older adults.

In addition to its greater bioavailability, krill oil also naturally contains astaxanthin and choline (from phosphatidylcholine), which are poorly consumed in the Western diet^(35)^. Astaxanthin is a powerful anti-inflammatory/antioxidant carotenoid that is considered a geroprotector^(38)^. Growing evidence suggests potential benefits of astaxanthin on several age-related conditions^(39,40)^ and modulation of inflammatory and neuropathic pain^(41,42)^. Choline is an essential nutrient that is involved in several biological processes relevant to aging, such as neurotransmission, cell signalling, lipid metabolism and homocysteine regulation^(43)^. Choline is also essential for skeletal muscle via modulation of fat and protein metabolism in muscle, decreasing fat synthesis and promoting muscle growth and function, as well as modulating inflammation, apoptosis and autophagy^(44)^. Yet, choline is consumed at suboptimal levels by the vast majority of older USA adults, with fewer than 3 % of adults aged 71 years or older consuming over the adequate intake level, suggesting widespread insufficiency^(45,46)^. In the USA, dietary choline is primarily obtained from animal-based foods such as meat, poultry, fish, dairy products and eggs, although it is also present in plant sources, including legumes, nuts and soy products. Notably, choline intakes below 50 % of the adequate intake level are associated with diminished strength and lean mass gains among older adults in a resistance exercise training programme^(47)^, as well as the risk of osteoporosis in the general older population^(48)^. Overall, krill oil could be a more effective approach to pain management in older adults than standard omega-3 supplementation and may provide additional health benefits relevant to aging populations.

While some studies have examined krill oil’s effects on pain, most have focused on middle-aged populations outside of the USA and lacked performance-based assessments of physical function^(49–52)^. All but one of these^(52)^ reported statistically significant reductions in self-reported pain intensity and interference compared with placebo^(49–51)^. In particular, Stonehouse *et al.* found that 4 g/d of Superba Boost™ krill oil (*n* 117) led to significant improvements in self-reported knee pain, stiffness and physical function compared with placebo (*n* 118) in middle-aged adults (40–65 years) with knee osteoarthritis^(49)^. In contrast, Laslett *et al.* found no significant difference in pain reduction with lower doses (2 g/d) of Superba Boost™ krill oil (*n* 130) compared with placebo (*n* 132) over 24 weeks in middle-aged and older adults with knee osteoarthritis^(52)^. Additionally, a recent randomised controlled trial in generally healthy older adults (≥ 65 years) found that 4 g/d of Superba Boost™ krill oil for 6 months significantly improved muscle function and size relative to placebo^(53)^.

We extend previous findings by evaluating the feasibility and acceptability of krill oil specifically on older adults with chronic musculoskeletal pain. Therefore, we designed a protocol for a pilot study to investigate the feasibility and acceptability of 4 g/d krill oil supplementation in older adults with chronic musculoskeletal pain and to evaluate its effects on plasma fatty acid profiles, inflammatory biomarkers, pain and mobility. This protocol adheres to Standard Protocol Items: Recommendations for Interventional Trials 2025 guidelines^(54)^. Table 1 shows the trial summary based on the WHO Registration Data Set.

**Table 1.** Trial summary Table 1 long description.

Title	Krill oil for pain and physical function in older adults
Brief title	Krill oil for pain in elders
Acronym	KOPE
Primary registry and trial identifying number	ClinicalTrials.gov NCT06580912
Secondary identifying numbers	P30AG028740, IRB202400581
Sources of monetary or material support	National Institute on Aging, Claude D. Pepper Older Americans Independence Center at the University of Florida (P30AG028740). Superba Boost™ krill oil and mixed vegetable placebo are manufactured and supplied free of cost by Aker BioMarine Antarctic USA LLC; Houston, TX, USA.
Primary sponsor	Claude D. Pepper Older Americans Independence Center University of Florida, USA info-aging@aging.ufl.edu
Central contact	Javier A. Tamargo, PhD, RDN University of Florida, USA (352) 273-5792 j.tamargo@ufl.edu
Countries of recruitment	USA
Condition(s) or focus of study	Chronic musculoskeletal pain
Interventions	
Krill oil	Intervention type: Drug Intervention name: Superba Boost Intervention description: 4 g per day of krill oil
Placebo	Intervention type: Dietary Supplement Intervention name: Mixed vegetable oil Intervention description: 4 g per day of mixed vegetable oil
Key eligibility criteria	Age eligibility: 60 years or older Sex eligibility: Both Accepts healthy volunteers: No Inclusion criteria: Provision of signed and dated informed consent form Stated willingness to comply with all study procedures and availability for the duration of the study Male or female, aged ≥ 60 years Exhibiting chronic musculoskeletal pain of the hip, knees, or lower back (> 3 months) Average pain ≥ 4 on a 0–10 numeric rating scale Exhibiting moderate mobility limitations (Short Physical Performance Battery score 4–10) Ability to take oral supplements and be willing to adhere to the supplementation regimen Agreement to adhere to Lifestyle Considerations throughout study duration Exclusion criteria: Any known coagulation or bleeding disorders Standing regimen of anticoagulants or full-dose aspirin Regular use of opioids or high-dose NSAID Taking medication known to affect muscle (e.g. steroids) Taking selective serotonin reuptake inhibitors Omega-3 supplementation within the past 3 months High consumption of fatty fish (> 2 servings/week) Habitual supplementation with other complementary medicines/supplements that may affect the study results, including St. John’s Wort Known allergy to seafood Clinically significant conditions: diabetes, severe CVD, seizure disorders, uncontrolled hypertension (> 150/90 mmHg at baseline), cancer, or cancer that has been in remission > 5 years History of atrial fibrillation or atrial flutter Dementia History of smoking, alcohol abuse, or illicit drug use Ambulatory impairments which would limit the ability to perform physical function tests Treatment with another investigational drug or other intervention within 3 months Planning a surgical procedure during the study period Planning to permanently leave the area during the study period
Study design	Study type: Interventional trial Allocation: Randomised Intervention model: Parallel group Primary purpose: Treatment Phase: Phase 2
Masking	Trial participants, Investigators, Outcome assessors
Date of enrolment	January 17, 2025
Target sample size	40
Recruitment status	Recruiting
Primary outcomes	Outcome: Feasibility and acceptability Timeframe: 3 months
Secondary outcomes	Outcome: Omega-3 Index, ω-6:ω-3 ratio, high-sensitivity C-reactive protein Timeframe: 3 months

## Objectives

### Primary objective

This study is designed as a randomised controlled pilot feasibility trial. Consistent with National Institutes of Health (NIH) guidance on pilot studies, the primary aim is not to determine efficacy, but to assess whether the intervention and trial procedures are feasible and acceptable in older adults with chronic musculoskeletal pain. Feasibility will be determined by meeting 80 % or more of the recruitment goal within 12 months, having less than 20 % attrition and achieving 70 % or greater adherence with the intervention. Acceptability will be determined through self-reported perceptions of convenience, taste and texture, appearance and smell, and side effects, as well as study experience and satisfaction.

### Secondary objectives

Secondary outcomes consist of the purported physiological changes of krill oil supplementation, determined by an increased ω-3 index, reduced ω-6:ω-3 ratio and reduction in inflammation. Due to small sample sizes, pilot studies cannot adequately provide evidence of safety, tolerability or effect sizes. As such, as an exploratory aim, we will evaluate changes in pain intensity and functional interference, and physical function, which will be the primary endpoints in a future, fully powered trial.

Because participant recruitment and study procedures were already underway during the peer review process, the protocol is described in the present tense to reflect its active conduct.

## Methods

### Trial design and setting

**Figure 1.**
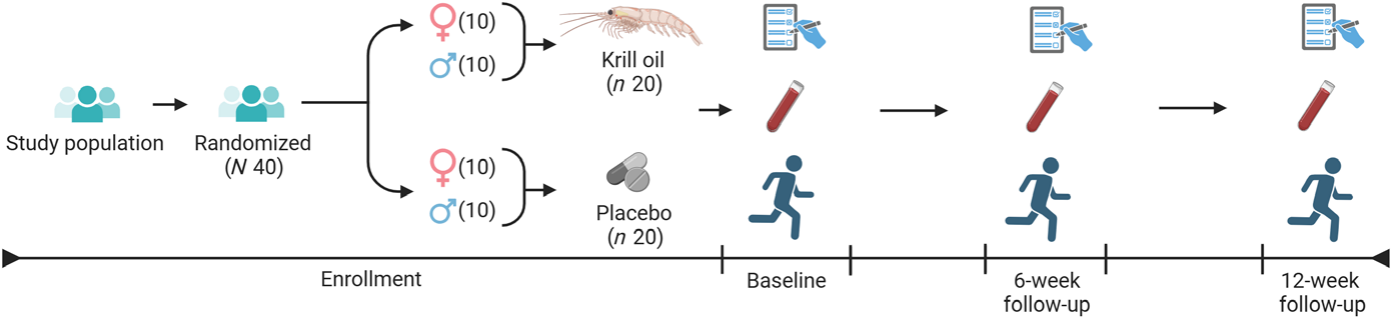
Krill oil for pain in elders study design. Forty participants were enrolled and randomised to receive either krill oil (*n* 20; ten females and ten males) or placebo (*n* 20; ten females and ten males), with study visits and blood sampling at baseline, 6 weeks and 12 weeks.

This pilot study will inform the feasibility and acceptability of future large-scale trials in older adults with chronic musculoskeletal pain. Feasibility is measured by metrics that indicate the ability to conduct a large-scale study, such as recruitment of the target population, retention of participants and adherence to the intervention^(55)^. The study will utilise a double-blind, randomised, placebo-controlled, repeated measures design (Figure 1). Eligible participants will be sex-stratified and randomised on a 1:1 ratio to either krill oil (*n* 20) or mixed vegetable oils placebo (*n* 20). The pilot study will test the protocol for a larger, fully powered trial by evaluating changes in plasma ω-3 index (%EPA + DHA in erythrocytes), the ω-6:ω-3 ratio and inflammation (e.g. high-sensitivity C-reactive protein) and obtain preliminary estimates of change in (1) pain intensity and functional interference and (2) mobility and physical function.

While participants were not involved in the initial design of this pilot study, we will involve them in the steering of the study, interpretation of the findings and design of larger future trials.

All research is conducted at the Pain Research & Intervention Center of Excellence and the Institute on Aging at the University of Florida.

### Eligibility criteria

The target population of this study is community-dwelling older adults with chronic musculoskeletal pain and moderate mobility limitations. Eligibility criteria are detailed in Table 1.

Briefly, individuals must be age 60 years or older, with moderate pain (≥ 4 on a 0–10 numeric rating scale) of the hip, knees, or lower back for more than 3 months, demonstrate mobility limitations and be willing and able to give informed consent. Due to the antiplatelet properties of ω-3s, we exclude anyone with a known coagulation or bleeding disorder, as well as those with a standing regimen of anticoagulants or full-dose aspirin. That said, current evidence does not support an increased risk of bleeding with ω-3 supplementation with or without the use of anticoagulants/antiplatelet drugs^(56)^. In an abundance of caution, we exclude those with a seafood allergy. Additionally, we exclude those who have supplemented with ω-3 PUFA in the last 3 months and those who report consuming fatty fish over 2 times per week. This cut-off is based on the current *Dietary Guidelines for Americans,* which recommends consuming at least 8 ounces (i.e. 2 servings) of seafood per week, providing ∼250 mg/d of EPA + DHA. Lastly, we exclude anyone with regular use of opioids or high-dose non-steroidal anti-inflammatory drugs within 30 d of randomisation.

### Interventions

The intervention group receives 4 g of krill oil (Superba Boost™ Krill, Aker BioMarine Antarctic USA LLC, Houston, TX, USA) in the form of four 1000 mg capsules of krill oil, once a day for 12 weeks. In total, participants receive 1288 mg/d EPA + DHA, 0·45 mg astaxanthin and 320 mg choline daily. Placebo capsules match the krill oil capsules in appearance, odour and taste, containing 1 g mixed vegetable oil, comprised of olive oil, corn oil, palm oil and medium-chain triglycerides in a ratio of 4:4:3:2, which contains approximately 31 % SFA, 46 % MUFA and 22 % PUFA, with no EPA or DHA. This mixture is similar to the fatty acid composition of the typical Western diet^(57,58)^. Within the context of the whole diet, the small amount of mixed fats consumed in the placebo is not expected to have any independent therapeutic effects. Participants are instructed to consume four capsules orally on one occasion with a meal, every day for 12 weeks. The product can be consumed at any time of the day; however, schedule consistency is recommended. Participants are encouraged to maintain their usual care, dietary habits, and physical activity levels.

Participants may discontinue the intervention at any time due to adverse events, personal preference or investigator discretion. Those individuals who discontinue the intervention will be encouraged to complete follow-up assessments.

### Modifications

No modifications to group allocation will be made after randomisation. However, participants may modify how they choose to consume the investigational product (e.g. time of day, with or without food, capsule spacing) to improve tolerability and adherence. This includes the option to gradually ramp up to the full daily dose of 4 capsules, as needed.

Discontinuation of the intervention may occur under the following conditions:

- Participant request, for any reason, including discomfort, inconvenience or perceived side effects.
- Adverse events that are judged by the study team to be possibly, probably or definitely related to the intervention and that pose a safety concern.
- Emergent medical conditions or new medication use that contraindicate continued supplementation.
- Investigator discretion, based on clinical judgement or safety monitoring findings.

Participants who discontinue the intervention will be encouraged to continue with all scheduled follow-up assessments to support intention-to-treat analyses.

### Adherence

Adherence is monitored through capsule counts and daily pill diaries, with adherence calculated as the percentage of capsules taken from those dispensed. Additionally, changes in the omega-3 index (%EPA + DHA in erythrocytes) will serve as an objective biomarker of adherence. Follow-up phone calls in weeks 1–3 and 9 will reinforce adherence, address concerns and remind participants of upcoming visits.

### Outcomes

Feasibility will be determined by meeting 80 % or more of the recruitment goal, having less than 20 % attrition and achieving 70 % or greater adherence with the intervention. Feasibility evaluation also includes assessing participant burden, including visit duration, assessment completion rates and participant feedback on study procedures. Acceptability will be assessed through self-reported perceptions of convenience, taste and texture, appearance and smell, and side effects, as well as study experience and satisfaction.

Secondary outcomes consist of the purported physiological changes of krill oil supplementation, determined by an increased ω-3 index, reduced ω-6:ω-3 ratio and reduction in inflammation. Due to small sample sizes, pilot studies cannot adequately provide evidence of safety, tolerability or effect sizes. As such, as an exploratory aim, we will evaluate changes in pain intensity and functional interference, and physical function, which will be the primary endpoints for a future, fully powered trial.

### Assessments

The schedule of events is shown in Table 2.

- Self-reported sociodemographic data, including age, sex, race and ethnicity, and income, are collected at the screening visit using standardised Common Data Elements.
- Adherence to the supplementation protocol is monitored through two methods: capsule counts and a daily pill diary. At each follow-up visit, the number of remaining capsules is recorded, and adherence is calculated as the percentage of capsules taken out of those dispensed.
- Anthropometrics: Height is measured only at baseline, while weight and waist circumference are assessed at every study visit, and BMI is calculated.
- Biological specimens are collected via whole blood samples. Fatty acid composition, including the omega-3 index and ω-6/ω-3 ratio, is assessed from dried blood spots using gas chromatography, following the validated method developed by OmegaQuant Analytics (Sioux Falls, sd, USA)^(59)^. The omega-3 index, defined as the percentage of EPA and DHA in erythrocytes, is considered a clinically relevant biomarker of ω-3 status and is highly responsive to dietary intake^(60,61)^. Absolute EPA and DHA concentrations will also be reported to enhance interpretability. High-sensitivity C-reactive protein is measured at every visit as part of the safety analyses.
  Participants are also asked to provide written consent for the optional storage and future use of blood specimens in an Institutional Review Board (IRB)-approved biobank. They are informed that all biospecimens and associated data stored in the biobank will be de-identified to protect their privacy. Participation in the biobank is entirely voluntary and does not affect eligibility or participation in the study.
- Pain assessments include a comprehensive pain history, including pain locations (via the Michigan Body Map)^(62)^, aetiology, duration, quality and aggravating or alleviating factors. The Graded Chronic Pain Scale-Revised assesses the severity and impact of chronic pain with five core items, including the 3-item Pain, Enjoyment and General Activity scale, which measures pain interference with daily life. The Graded Chronic Pain Scale-Revised categorises individuals into three groups: no chronic pain, low-impact chronic pain and high-impact chronic pain^(63)^. The Western Ontario and McMaster Universities Osteoarthritis Index evaluates pain, stiffness and physical function in individuals with musculoskeletal conditions, particularly osteoarthritis. It includes three subscales for pain, stiffness and physical function^(64)^. The PainDETECT questionnaire screens for neuropathic pain^(65)^. Movement-evoked pain refers to the pain during activity, which is a primary driver of musculoskeletal pain^(66,67)^. Movement-evoked pain is assessed immediately following each physical function task using a 0 to 100 numerical rating scale^(68)^. The Patient Global Impression of Change assesses an individual’s overall perception of change in their condition following treatment with responses ranging from ‘very much improved’ to ‘very much worse’^(69)^.
- Disability is measured using the Pepper Assessment Tool for Disability, a 23-item instrument that evaluates five domains of function in older adults: mobility, transferring, upper extremity function, activities of daily living and instrumental activities of daily living^(70,71)^.
- Physical function is assessed using the Short Physical Performance Battery, which includes tests of balance, gait speed and lower extremity strength^(72,73)^. Handgrip strength is measured using a handheld dynamometer^(74)^, and the 6-minute walk test evaluates functional capacity and endurance^(75)^.
- Dietary intake is assessed using the Diet History Questionnaire III, a web-based, self-administered FFQ that captures intake of 135 food and beverage items and 26 dietary supplements over the past month.
- Cognitive function is evaluated using the montreal cognitive assessment, a brief screening tool for global cognitive function^(76)^, and the NIH Toolbox Cognition Battery, which includes seven tests covering six cognitive domains. Composite scores are generated for fluid, crystallised and global cognition^(77,78)^. Alternate versions of the montreal cognitive assessment are used at each visit to minimise retest effects.
- The acceptability of the intervention and trial design is assessed with the Medicine Acceptability Questionnaire^(79)^ and the Study Participant Feedback Questionnaire^(80)^.
- Mood/affective factors are assessed with the Fear Avoidance Beliefs Questionnaire^(81)^, Pain Self-Efficacy Questionnaire^(82)^, Perceived Stress Scale^(83)^ and the Geriatric Depression Scale-Short Form^(84)^. The Brief Pittsburgh Sleep Quality Index is used to assess sleep quality^(85)^. Quality of life is assessed with the EuroQol 5 Dimension 5 Level^(86)^.

**Table 2.** Schedule of activities Table 2 long description.

	Study period					
	Enrolment		Post-randomisation			Close-out
	Screening	Baseline	Q1	Midpoint	Q3	Final
Timepoint (weeks)	(–2 to 1)	(1)	(3±1)	(6±1)	(9±1)	(12±1)
Enrolment						
Informed consent	X					
Randomisation		X				
Interventions						
Intervention Group A		X	X	X	X	X
Intervention Group B		X	X	X	X	X
Assessments						
Sociodemographic questionnaires	X					
Medical history & medications/update	X	X		X		X
Vital signs (blood pressure, pulse, temperature)	X	X		X		X
Height	X					
Weight	X	X		X		X
Waist circumference		X		X		X
Pain history	X					X
Graded Chronic Pain Scale-Revised	X	X		X		X
Adverse events			X	X	X	X
Short Physical Performance Battery	X			X		X
Handgrip strength		X				X
6-minute walk test		X				X
Capsule count		X		X		X
Whole blood collection		X		X		X
• Dried blood spot (OmegaQuant)		X		X		X
• Comprehensive metabolic panel, complete blood count, lipids		X		X		X
• Inflammation (high-sensitivity C-reactive protein)		X				X
• Biorepository (optional)		X				X
Western Ontario and McMaster Universities Arthritis Index		X		X		X
PainDETECT		X				X
Patient Global Impression of Change			X	X	X	X
Medicine Acceptability Questionnaire				X		X
Pepper Assessment Tool for Disability		X		X		X
Fear Avoidance Beliefs Questionnaire		X				X
Pain Self-efficacy Questionnaire		X				X
EuroQol 5 Dimension 5 Level		X				X
Brief Pittsburgh Sleep Quality Index		X		X		X
Perceived Stress Scale		X				X
Geriatric Depression Scale-Short Form		X				X
Diet History Questionnaire, version III		X				X
Montreal Cognitive Assessment	X			X		X
National Institutes of Health Toolbox Cognition		X				X
Study Participant Feedback Questionnaire						X

### Sample size

This pilot study is designed as a small-scale trial to inform the design and implementation of future large-scale studies evaluating the effectiveness of krill oil supplementation in older adults with chronic musculoskeletal pain. A total of 40 participants will be enrolled and randomised in a 1:1 ratio to receive either krill oil (*n* 20) or placebo (*n* 20) for 12 weeks. Randomisation is stratified by sex, resulting in balanced representation within each group (ten males and ten females per arm). Accounting for an anticipated 20 % attrition rate, we expect approximately sixteen participants per group to complete the study, which is sufficient to assess feasibility and is also within methodological recommendations for pilot randomised trials in which anticipated effects are in the small-to-moderate range^(87)^.

### Recruitment

Recruitment began in January 2025 and will continue through December 2025, or until the target enrolment of 40 participants is achieved. Participants are recruited from the areas surrounding the University of Florida in Gainesville, as well as from the broader North and Central Florida regions. Recruitment strategies include outreach through the University of Florida Pepper Registry, direct mail campaigns, social media advertising and flyer distribution in clinical and community settings.

### Randomisation

The random allocation sequence was generated by Dr. Yenisel Cruz-Almeida, Co-Investigator, using PASS 2024 (NCSS, LLC, Kaysville, Utah, USA). A block randomisation approach with randomly permuted block sizes of two and four was used, stratified by sex to ensure balanced group sizes and control for sex-related differences in outcomes. The allocation sequence was imported into Research Electronic Data Capture (REDCap), which serves as the central platform for randomisation. REDCap assigns participants to intervention groups automatically upon enrolment, using the preloaded randomisation table. This process ensures allocation concealment, as the sequence is not visible to the study staff responsible for enrolling participants. The principal investigator and other personnel who interact with participants do not have access to the randomisation sequence.

### Blinding

This is a double-blind trial in which participants, interventionists, outcome assessors and data analysts are all blinded to group assignments. The Co-Investigator who generated the randomisation sequence (YCA) also performed the statistical analyses and was necessarily unblinded to group assignments; however, YCA has no involvement in participant recruitment, enrolment, or clinical assessments. Groups are labelled as A and B to maintain blinding. Additionally, access to fatty acid analysis results from OmegaQuant is restricted after baseline to prevent unblinding through observed changes in omega-3 levels. Unblinding is only permitted when necessary for participant safety and must be approved and documented by the principal investigator.

### Retention

All participants are asked to attend one screening visit, one baseline visit and two follow-ups at 6 and 12 weeks. Retention is actively monitored and discussed by the Principal Investigator and study coordinators during weekly meetings, where missed visits, scheduling difficulties or other concerns are reviewed and addressed with action plans. Participants who cannot be reached after four contact attempts or fail to attend their screening visit are deemed ineligible. To support retention, staff schedule upcoming visits during current appointments, send email reminders and place reminder calls on the business day prior. Missed visits trigger immediate follow-up calls and tailored efforts to re-engage participants within the scope of clinic services.

### Data management

All data collection is conducted by trained clinical trial staff under the supervision of the Principal Investigator. Data are collected electronically via Research Electronic Data Capture, a secure, web-based application that is compliant with the USA. Food and Drug Administration 21 CFR Part 11 standards. To ensure data accuracy and integrity, the Principal Investigator conducts weekly monitoring of recruitment, retention, adverse events and 100 % data verification. All data handling follows written Standard Operating Procedures to ensure compliance with Good Clinical Practice. Participant data are de-identified and stored using unique study identification numbers. Personally identifiable information is stored separately on a secure server maintained by the University of Florida, with restricted access limited to authorised study personnel. All records are retained in accordance with institutional and regulatory requirements.

### Statistical approach

Feasibility and acceptability will be determined as (1) recruitment of at least 80 % of the target sample (*n* 32), (2) attrition rate ≤ 20 % and (3) adherence rate ≥ 70 % (based on capsule counts and intake diaries). Changes in fatty acid profile (i.e. omega-3 index) will be analysed using mixed-effects linear regression models with fixed effects for treatment group, time (as categorical) and a group-by-time interaction. While there is currently no guidance on what may constitute a minimal clinically meaningful change in plasma ω-3 index, current evidence supports that an ω-3 index ≥ 8 % may be protective against CVD, whereas an index of ≤ 4 % indicates increased risk^(88)^. Given the pilot nature of the trial, analyses will emphasise estimation of between-group differences and corresponding 95 % CI rather than formal hypothesis testing. Estimates of variability and feasibility metrics will be used to inform outcome selection and design considerations for future trials. Changes in pain intensity, pain interference and physical function will be analysed using mixed-effects linear regression models, with fixed effects for treatment group, time (as categorical) and a group-by-time interaction, adjusting for age, sex and BMI. A random intercept will be included for each participant, assuming an independent covariance structure. A 20 % pain reduction on a 0–10 numeric rating scale is considered a clinically significant pain reduction^(89)^. A one-point change in the Short Physical Performance Battery and a 50-m increase in the 6-minute walk test are considered clinically meaningful changes for older adults^(90)^. All analyses will follow an intent-to-treat approach, including all randomised participants in the groups to which they were assigned, regardless of adherence. A per-protocol analysis may also be conducted as a sensitivity analysis, including only participants with ≥ 70 % adherence. Missing data will be addressed using maximum likelihood estimation within the mixed-effects models, which accommodates data missing at random. Sensitivity analyses may be conducted using multiple imputations if warranted.

### Trial and data monitoring

A Data and Safety Monitoring Board (DSMB) has been established to oversee the safety and progress of all studies conducted within the University of Florida’s Claude D. Pepper Older Americans Independence Center. This committee is composed of three experienced professionals and operates independently from the study sponsor, the National Institute on Aging and from the investigative team. All members are free of conflicts of interest. The DSMB is responsible for reviewing adverse events, monitoring study conduct and making recommendations regarding the continuation, modification or early termination of the trial.

In addition to DSMB oversight, trial conduct will be monitored weekly by the Principal Investigator. Monitoring activities include tracking participant recruitment and retention, reviewing adverse events, verifying 100 % of data entries and ensuring adherence to the protocol. Any serious adverse events that may be related to the intervention will be reported to the NIH within 48 h and to the IRB, the Pepper Center DSMB and the USA. Food and Drug Administration within five business days, as applicable. The study may be stopped early if adverse effects significantly alter the risk-benefit ratio, if recruitment or retention becomes infeasible, if new information emerges that necessitates early termination or if the DSMB recommends discontinuation based on safety concerns. Only the DSMB and designated safety monitors will have access to unblinded interim safety data.

While no formal interim analyses are planned, the DSMB reviews safety data biannually and may recommend early termination if significant safety concerns arise.

### Safety/Harms

Adverse events in this study are defined as any adverse medical occurrences experienced by participants during the trial, regardless of whether they are considered related to the intervention. These are assessed systematically through a combination of clinical evaluations and participant self-reporting. Vital signs (i.e. blood pressure, pulse and temperature) are measured at each study visit to monitor acute physiological changes and ensure safety to proceed with study procedures. Blood samples are collected at baseline and subsequent in-person visits to assess comprehensive metabolic panel, complete blood count, lipid panel and high-sensitivity C-reactive protein. If initial results are inconclusive or further analysis is warranted, participants are asked to return for additional blood drawings. Adverse events are actively monitored by querying participants during every follow-up interaction, including quarterly phone calls and in-person visits. Medical history is reviewed at screening and updated at each visit to identify any new or worsening conditions.

### Protocol amendments

All changes to the protocol, including modifications to eligibility criteria, study procedures, outcome measures or statistical analysis plans, will be submitted to the University of Florida IRB and the USA. Food and Drug Administration as protocol amendments under the active IND. Amendments will be clearly labelled and include a summary of changes, rationale and updated protocol documents. Changes will not be implemented until IRB approval is obtained, except when necessary to eliminate immediate hazards to participants, in which case the IRB and Food and Drug Administration will be notified as soon as possible. All amendments will be documented in the protocol amendment history table and communicated to study staff, the DSMB and participants as appropriate.

### Informed consent process

Informed consent is obtained in-person by the Principal Investigator or IRB-approved study staff prior to any study-specific procedures. Participants receive an IRB-approved consent form and are given time to review it, ask questions and discuss participation with family. The study is explained in clear, accessible language, including its purpose, procedures, risks and voluntary nature. Participants are informed they may withdraw at any time without penalty. Consent is documented with a signed form, and participants receive a copy for their records.

Participants are also asked to provide written consent for the optional storage and future use of blood specimens in an IRB-approved biobank. They are informed that only de-identified biospecimens and associated data are stored in the biobank to protect their privacy. Participation in the biobank is entirely voluntary and does not affect eligibility or participation in the study.

### Confidentiality

Participant confidentiality is strictly maintained by the study team and sponsor, including all clinical data and biological samples. Study records are stored securely and are only accessible to authorised personnel. Identifiable information will not be shared without prior written approval from the sponsor. Participants are identified by unique study ID, and de-identified data are stored on secure, access-controlled systems for analysis and reporting. All research activities are conducted in private settings. Regulatory authorities, IRB representatives and study monitors may inspect study records as required. Records will be retained in accordance with IRB, institutional and sponsor policies.

### Access to data

Access to the final trial dataset will be restricted to the Principal Investigator, co-investigators and authorised study staff at the University of Florida. There are no contractual agreements with sponsors or external parties that restrict the investigators’ access to the full dataset or their ability to analyse and publish the results.

### Dissemination policy

This study will be conducted in accordance with the NIH Public Access Policy, which ensures public access to the published results. Every effort will be made to publish study findings in peer-reviewed scientific journals and to present results at relevant scientific conferences. Final peer-reviewed manuscripts will be submitted to PubMed Central upon acceptance for publication. The study will also adhere to the NIH Data Sharing Policy and the Policy on the Dissemination of NIH-Funded Clinical Trial Information. The trial is registered on ClinicalTrials.gov, and summary results will be submitted in accordance with the Clinical Trials Registration and Results Information Submission rule.

Results will be shared with participants in a lay summary format upon study completion.

### Reproducible research

The full trial protocol and/or de-identified datasets, along with the data dictionary and statistical code, may be made available to qualified researchers upon request, after completion of the primary endpoint. Requests should be made to the Principal Investigator.

## Discussion

Chronic musculoskeletal pain and mobility limitations are highly prevalent and debilitating conditions among older adults, contributing significantly to functional decline, loss of independence and reduced quality of life^(1,2,4,5,10–13)^. Given the limitations and risks associated with pharmacological treatments in this population^(15,18)^, there is a growing interest in non-pharmaceutical interventions that are both safe and effective^(7,15)^. Omega-3 PUFA supplementation has emerged as a promising candidate, with krill oil offering unique advantages due to its superior bioavailability and additional astaxanthin, phospholipids and choline^(35)^.

To date, most studies evaluating the effects of krill oil on pain and physical function have been conducted in middle-aged populations and outside the USA.^(48–51)^ These studies often lack representation of older adults, the population most affected by chronic musculoskeletal pain and mobility limitations. The current study is designed to address this gap by evaluating the feasibility and acceptability of a krill oil supplementation intervention, specifically in older USA adults with chronic musculoskeletal pain. This trial targets a high-risk group and incorporates a comprehensive characterisation of pain and validated geroscience-based physical function assessments.

As a pilot study, several limitations must be acknowledged. Due to the relatively short intervention period (12 weeks) and small sample size (*n* 40), any findings should be interpreted with caution. Generalisability may also be constrained by the study’s eligibility criteria, which may not fully reflect the broader population of older adults with chronic musculoskeletal pain. For example, the exclusion of individuals using opioids, high-dose NSAID, anticoagulants or recent omega-3 supplementation may limit generalisability, as these are commonly used by older adults. However, the randomised, double-blind, placebo-controlled design strengthens internal validity and helps mitigate potential biases. The study is also strengthened by its comprehensive and multidimensional assessment strategy. This includes a variety of well-validated self-report pain measures, experimental pain assessment through movement-evoked pain, and performance-based physical function tests grounded in geroscience.

## Conclusion

This pilot study will provide data on the feasibility and acceptability of krill oil supplementation in older adults with chronic musculoskeletal pain and mobility limitations, as well as preliminary estimates of change in clinical outcomes to inform the design of future definitive trials. By targeting a population that is particularly susceptible to these conditions, the study addresses a significant and growing public health concern. The data generated will offer valuable guidance for the development of future randomised controlled trials with larger samples and longer follow-up periods, ultimately contributing to the development of non-pharmacological strategies to support health, longevity, and independence in older adults with chronic musculoskeletal pain.

## Table 1. Long description

A table summarizing a clinical trial on krill oil for pain in older adults. The table includes details such as the trial title, brief title, acronym, primary and secondary registry identifying numbers, sources of monetary or material support, primary sponsor, central contact, countries of recruitment, condition or focus of study, interventions, key eligibility criteria, study design, masking, date of enrolment, target sample size, recruitment status, primary and secondary outcomes. The table has multiple rows and columns, providing a comprehensive overview of the trial’s structure and objectives.

Navigate back to Table 1..

## Table 2. Long description

The table outlines the schedule of activities for a study period, divided into enrolment, post-randomisation, and close-out phases. It includes timepoints in weeks, interventions for Group A and Group B, and various assessments such as sociodemographic questionnaires, medical history updates, vital signs, height, weight, waist circumference, pain history, graded chronic pain scale-revised, adverse events, short physical performance battery, handgrip strength, 6-minute walk test, capsule count, whole blood collection, Western Ontario and McMaster Universities Arthritis Index, PainDETECT, patient global impression of change, medicine acceptability questionnaire, pepper assessment tool for disability, fear avoidance beliefs questionnaire, pain self-efficacy questionnaire, EuroQol 5 Dimension 5 Level, Brief Pittsburgh Sleep Quality Index, Perceived Stress Scale, Geriatric Depression Scale-Short Form, Diet History Questionnaire, version III, Montreal Cognitive Assessment, National Institutes of Health Toolbox Cognition, and Study Participant Feedback Questionnaire. The table has 26 rows and 6 columns, detailing the specific activities and assessments conducted at each study visit.

Navigate back to Table 2..
